# Monocyte-to-HDL ratio and non-HDL cholesterol were predictors of septic shock in newborns

**DOI:** 10.1016/j.clinsp.2022.100111

**Published:** 2022-11-08

**Authors:** Fernanda Andrade Macaferri da Fonseca, Aline Paulino Espósito, Maria Helena Baptista Nunes da Silva, Valéria Sutti Nunes, Patricia Miralda Cazita, Guilherme Silva Ferreira, Maria Esther Jurfest Rivero Ceccon, Werther Brunow de Carvalho, Magda Carneiro-Sampaio, Patricia Palmeira

**Affiliations:** a Departamento de Pediatria, Faculdade de Medicina FMUSP, Universidade de Sao Paulo, Sao Paulo, SP, BR.; b Instituto da Criança, Hospital das Clinicas HCFMUSP, Faculdade de Medicina, Universidade de Sao Paulo, Sao Paulo, SP, BR; c Laboratorio de Lipides (LIM10), Hospital das Clinicas HCFMUSP, Faculdade de Medicina, Universidade de Sao Paulo, Sao Paulo, SP, BR; d Laboratorio de Pediatria Clinica (LIM36), Hospital das Clínicas HCFMUSP, Faculdade de Medicina, Universidade de Sao Paulo, Sao Paulo, SP, BR

**Keywords:** Late-onset neonatal sepsis, Septic shock, Lipoproteins, Monocyte-to-HDL ratio, ***:*** D, Day, CT, Cholesterol, TG, Triglyceride, VLDLc, very-low-density lipoprotein, LDLc, low-density lipoprotein, HDLc, high-density lipoprotein, (M/H), monocyte-to-HDL ratio, NICU, neonatal intensive care unit, I/T, immature/total neutrophil ratio, ANC, absolute neutrophil count, CRP, C-reactive protein, OR, odds ratio, CI, confidence interval, Delta (Δ), D0 - D3

## Abstract

**Background:**

The association between lipoprotein levels and late-onset neonatal sepsis has shown controversial results. The aims are to assess lipid profile, cytokines, and Monocyte-to-HDL (M/H) ratio as diagnostic and prognostic markers for late-onset neonatal sepsis.

**Methods:**

This prospective study included 49 septic neonates and 17 controls. Cholesterol (CT), Triglyceride (TG), Very-Low-Density (VLDLc), Low-Density (LDLc), and High-Density Lipoproteins (HDLc) were measured at admission (D0) and on days 3, 7 and 10 to evaluate septic shock outcomes. Cytokines and monocytes were evaluated by flow cytometry.

**Results:**

Septic newborns showed higher IL-6 and IL-8 at D0 and CT levels on D7 and on D10, which also presented higher TG, VLDLc and non-HDL cholesterol concentrations than controls. The septic shock group (n = 22) revealed a higher number of male subjects, CRP, IL-6, IL-8 and IL-10 levels, while lower TG, HDLc, monocyte numbers and M/H ratio at admission compared to the non-shock group (n = 27). M/H ratio and non-HDL cholesterol on D0 were risk factors for septic shock (OR = 0.70, 0.49‒0.99; OR = 0.96, 0.92‒0.99, respectively). Decreasing levels from D0 to D3 of CT (OR = 0.96, 0.93‒0.99), VLDLc (OR = 0.91, 0.85‒0.98), and non-HDL cholesterol (OR = 0.92, 0.87‒0.98) were also predictors of septic shock.

**Conclusions:**

Lower M/H ratios and non-HDL cholesterol at admission and decreasing levels of cholesterol, VLDLc and non-HDL cholesterol during a hospital stay are associated with the development of septic shock in newborns with late-onset neonatal sepsis.

## Highlights

• Profound changes in lipid profile have been described in septic patients.
• Lipid profile was equivalent between septic and control groups.
• Septic shock group showed lower TG, HDLc, monocytes and M/H ratio and higher CRP, IL-6, IL-8 and IL-10 levels at admission.
• All lipid variables decreased from D0 to D3 in the shock group.
• M/H ratio and non-HDL cholesterol were predictors of shock in septic newborns.

## Introduction

Sepsis is a major cause of morbidity and mortality in Newborns (NB) with high incidence despite advances in intensive care. It is estimated that 1,3 million NB suffer from sepsis globally each year, with an incidence of 2,824 per 100,000 live births and mortality of 17.6%.^1^^,^^2^ Early diagnosis of neonatal sepsis remains a major challenge due to the non-specific and subtle clinical signs and symptoms of the disease, and early recognition and prompt treatment are crucial to improve outcomes for neonates with severe sepsis and septic shock.^3^ Among the diagnostic tests currently employed, isolations by peripheral blood culture (blood cultures), cerebrospinal fluid, or urine still constitute the reference tests for sepsis diagnosis. However, microorganism growth requires significant time to define the diagnosis, with high specificity, but low sensitivity. Currently, the best biomarkers combination for diagnosing neonatal sepsis are IL-6 and IL-1ra, CRP, procalcitonin, and hematological indexes,^4^^,^^5^ however, there is no marker that can reliably differentiate infected from uninfected children.

Lipoproteins and lipids, which have direct immunomodulatory properties, bind, and neutralize toxic bacterial substances and have been identified as candidates for biomarkers of infection. Lipoproteins can negatively regulate the inflammatory response, being considered protective factors during sepsis.^6^ Some studies have shown the ability of LDLc, VLDLc, and particularly, HDLc to potentially modulate the acute inflammatory response through the sequestration of Lipopolysaccharide (LPS), as well as lipoteichoic acid from Gram-negative and positive microorganisms, respectively.^7^

Several studies reported low HDLc levels in established adult septic shock.^7^^,^^8^ More specifically, low HDLc during septic shock is generally associated with increased hospital mortality.^9^ In late-onset neonatal sepsis, TG levels were found to be lower than those of healthy controls, and low serum apo-lipoprotein A levels would be a predictive marker for diagnosis.^10^

It was shown that HDLc molecules counteract the migration of macrophages who ingested oxidized LDLc and other lipids through their scavenger receptors and remove cholesterol from these cells. In addition, blood monocyte count is predictive of new atherosclerotic plaque development.^11^ Alternatively, individuals with higher HDLc levels had 42% decreased odds of being in the top quartile of monocyte count. In this context, it was reported that high circulating monocyte counts and reduced HDLc concentrations, both used to define the monocyte-to-HDL (M/H) ratio, are a recently defined parameter in the diagnosis of cardiovascular diseases and may predict adverse outcomes in patients with chronic kidney disease.^12^^,^^13^

However, the prognostic value and relation to the disease outcome of lipoproteins in neonates with late-onset sepsis has not yet been elucidated. The aim of this study was to investigate the use of cholesterol, TG, lipoproteins, and monocyte-to-HDL (M/H) ratio as additional tools for the diagnosis and prognosis of late-onset neonatal sepsis.

## Materials and methods

### Research design

This was a prospective study conducted in the Neonatal Intensive Care Units (NICU) of the Instituto da Criança, Hospital das Clínicas da Faculdade de Medicina da Universidade de São Paulo (HCFMUSP), and in the NICU of the Hospital Ipiranga from April/2017 to February/2020. The study was approved by the Research Ethics Committee of the participating institutions (CAAE: 53495516.6.0000.0068).

The total sample included 66 late-preterm/full-term newborns (gestational age ≥34 weeks) who were evaluated for late-onset sepsis (symptoms onset after 72 hours up to 30 days of life) at the time of admission or during hospitalization, which led to the start of antibiotic therapy. The septic group included neonates with proven infection (positive blood [BD Bactec™]) or with clinical sepsis, defined as negative cultures but clinical and laboratory evidence of sepsis characterized as two signs/symptoms suggestive of sepsis, along with two altered laboratory parameters in the ancillary exams performed to investigate the potential infection. All the patients who were initially hospitalized with suspected sepsis, but the diagnosis of sepsis was not supported by clinical or laboratory findings were included in the control group. Some of these controls presented only one clinical and/or one laboratory parameter, not fulfilling the sepsis criteria.

Septic patients were classified according to the criteria reported by Goldstein et al.^14^ maintained in the Surviving Sepsis Campaign in 2020,^15^ which kept the Goldstein et al. concept for neonates. So, for sepsis evaluation, at least, two of the following clinical conditions were considered: Thermal instability, that is, hypothermia or hyperthermia (axillary temperature < 36°C and > 38°C, respectively); Cardiovascular compromise, that is, heart rate < 90 and > 180 beats per minute, pallor, decreased perfusion (capillary refill of 3 seconds or cold extremities), or hypotension, and mean arterial pressure (mmHg) (< 10 and > 95 percentiles); Respiratory compromise, that is, bradypnea or tachypnea (respiratory rate ≤ 20 and > 50 breaths per minute, according to post-natal age), or increased apnea (cessation of respiration for 20 seconds, occurring at a rate of 2 times per hour), severe apnea (any single episode requiring positive pressure ventilation), increased ventilatory support (with no other obvious cause, e.g., pneumothorax); Gastrointestinal tract compromise, that is, feeding intolerance (increased gastric residuals of 30% of food volume in 2 feedings within 24 hours).

In addition, two altered laboratory parameters of the following were included: White blood cell count; Absolute Neutrophil Count (ANC); Immature neutrophil count; I/T ratio; Platelet count; C-Reactive protein levels (> 10 mg/L); Direct bilirubin (> 2 mg/dL); Lactate (≤ 4.5 and ≥ 28.8 mg/dL).^16^

Septic shock was defined as severe infection leading to cardiovascular dysfunction (including hypotension, need for treatment with a vasoactive medication, or impaired perfusion). Hypotension was defined as a mean blood pressure value fifth percentile of gestational-age- and postnatal-age-dependent blood pressure values.^15^

To perform a serial follow-up, blood was collected from all septic newborns in, at least, three moments: at the time of initial laboratory evaluation (D0); within 72 hours (D3); and 7 days after initiation of treatment (D7). In case of clinical deterioration of the newborn and/or prolonged treatment, a new blood sample was collected on day 10 after diagnosis (D10). For ethical reasons, in control newborns, only one blood withdrawal was performed.

The neonates were monitored during treatment to observe the appearance of septic shock and/or death. Then, the neonates were regrouped according to the progression of their outcomes, those who developed shock and/or death (Shock group), and those who did not develop shock and survived (non-shock group). Attributable early mortality was defined as death occurring within 28 days of life.

Gestational ages, delivery type, birth weight, gender, age at sepsis evaluation, weight at sepsis evaluation, duration of mechanical ventilation, vasopressor use, death, and microorganisms isolated were recorded.

All patients (including controls) were administered antibiotics initially according to standard protocols from the NICU and antibiotic treatments were discontinued in patients without sepsis, after 72 hours. However, in patients with culture-proven or clinically diagnosed sepsis, antibiotic therapies were given for, at least, 7 days, for a maximum of 21 days depending on the bacteria detected or patient follow-up.

Exclusion criteria applied for septic and control groups were diagnosis of congenital infections, inborn errors of metabolism, and chromosomal abnormalities.

Blood samples were collected from a peripheral vein in EDTA-coated tubes for immunophenotyping and to obtain plasma for lipoproteins analysis, and in special clot activator tubes for serum separation. Plasma and serum samples were aliquoted and stored at -80°C.

IL-1β, IL-8, IL-6, IL-10, IL-12, and TNF-α concentrations were measured in serum samples using the cytometric bead array technique (Becton Dickinson, BD Biosciences, San Jose, CA, USA), according to the manufacturer's instructions.

### Immunophenotyping

The total leukocyte number obtained from peripheral blood was determined with an automatic counter (Sysmex XP-300, Sysmex Corporation, Kobe, Japan). To analyze monocytes, 1 × 10^6^ leukocytes were stained for 30 minutes with fluorochrome-conjugated monoclonal antibodies against CD14 and HLA-DR. After two-step washes, the cells were resuspended in BD FACSFlow™ (BD Biosciences, San Jose, CA, USA) and immediately analyzed. A total of 10,000 events in the monocyte gate were acquired with a BD LSRII Flow Cytometer™ (BD Biosciences) using the BD FACSDiva software (Becton Dickinson), and the analysis was performed using FlowJo software (Tree Star, Ashland, OR, USA). For the analysis, a gate was set based on the forward- and side-scatter characteristics (FSC-A  ×  SSC-A), and then, monocytes were identified as CD14^+^HLA-DR^+^ cells. Data are presented as absolute numbers, which were calculated from the complete leukocyte counts. The monocyte to HDL ratio was calculated for each sample as the ratio of the absolute monocyte count to the HDL concentration.

### Plasma lipids and lipoproteins

Plasma total cholesterol and triglyceride were determined by an enzymatic-colorimetric method using Labtest kits (Labtest Diagnostica, MG, Brazil) according to the manufacturer's instructions. Plasma lipoproteins as VLDLc, LDLc, HDLc were separated by Fast Protein Liquid Chromatography (FPLC) in AKTA Purifier liquid chromatography system (Amersham-Pharmacia Biotech., Uppsala, Sweden). Plasma (100 µL) was injected on HR 10/30 Superose 6 column (GE Healthcare 17-5172-01), and elution occurred at a constant flow rate of 0.5 mL/min with Tris buffer (10 Mm Tris, 150 mM NaCl, 1 mM EDTA and 0.03% NaN3, pH 7.0). Fractions of 0.2 mL were collected in 96 well plates using the fraction collector.

Cholesterol and triglyceride from 60 fractions were measured by an enzymatic-colorimetric method using Labtest kits (Labtest Diagnostica, MG, Brazil) according to the manufacturer's instruction in order to identify the peaks corresponding to VLDLc, LDLc and HDLc.^17^^,^^18^

### Statistical analyses

Statistical analyses were performed using the software Minitab 19. Categorical variables were presented as frequencies and evaluated by Person's Chi-Square test. Continuous variables were presented as mean ± SD or median (Q1 – Q3) and analyzed by non-paired Student *t*-tests or Mann-Whitney, according to data distribution. A p < 0.05 was considered statistically significant.

Normality distribution was checked by using the Anderson-Darling test or Shapiro-Wilk test. Correlations were tested between continuous variables with Pearson or Spearman rank correlation coefficients. In order to avoid type I error, Bonferroni's correction was used to verify the differences between sepsis and control groups over time. Thus, the threshold of statistical significance considered in these analyses was p < 0.0125. Binary logistic regressions (simple and multiple) were presented as Odds Ratios (ORs) and their respective 95% Confidence Interval (95% CI) and were run to determine the association between biochemical variables and the risk of septic shock development. In multiple logistic regressions, gender was included in the model as a covariate due to its strong association with the development of septic shock. The calibration of the model was assessed by the Hosmer-Lemeshow goodness-of-fit test (p > 0.05). Receiver Operating Characteristic (ROC) curve analysis was performed using the Youden index to select the optimal cut-off values.

## Results

### Demographic, clinical, and laboratorial characteristics of the neonates

Of the 49 septic infants enrolled in the study, 30 had positive blood cultures: 11 cases were due to Gram-negative organisms; 13 cases were due to Gram-positive organisms; 6 were due to fungi organisms; and 19 infants had clinical sepsis, and an additional 17 newborns were enrolled in the control group. The clinical and laboratorial characteristics of the newborns at D0 are summarized in Table 1. Of the 49 septic patients, sixteen presented thermal instability (32.7%), forty-six presented cardiovascular compromises (93.9%), forty-three presented respiratory compromises (87.8%), twenty-two presented with gastrointestinal tract compromises (44.9%) and forty-four presented altered laboratory parameters (89.8%), which are described in Table 1.

**Table 1:** Main demographic, clinical and laboratory findings of septic and control groups.

Characteristic	Sepsis (n = 49)	Controls (n = 17)
Sex (Male/Female)	34/15	9/8
Delivery type (C-section/vaginal)	31/18	9/8
Gestational age (week)	37 2/7 (35 6/7‒38 1/7)	36 6/7 (35‒39 4/7)
Birth weight (g)	2666 (±565)	2919 (±632)
Age at sepsis evaluation (days)	17 (11‒26)^b^	10 (4‒18)
Weight at sepsis evaluation (g)	2967 (±495)	2966 (±508)
Respiratory support (mechanical ventilator/CPAP) (%)	15 (30)	‒
Duration of mechanical ventilation (days)	13 (±8)	‒
Vasopressor use, n (%)	22 (45)	‒
White blood cell count (/uL)	14640 (10040‒18403)	13080 (10955‒16860)
Absolute neutrophil count (/uL)	6690 (4923‒12955)	5930 (4160‒10995)
Immature neutrophils (/uL)	335 (0‒680)^c^	0 (0‒26)
Immature/total neutrophil ratio	0.04 (0‒0.08)^b^	0 (0‒0.004)
Platelet count (× 10^3^/uL)	317 (200‒456)	330 (257‒453)
C-reactive-protein (mg/L)	12.6 (4.6‒75.8)^d^	1.1 (0.6‒2.4)
Direct bilirubin (mg/dL)	1.1 (0.4‒1.7)^a^	0.4 (0.2‒0.7)
Lactate (mg/dL)	18 (13‒25)	19 (15‒19)
Septic Shock, n (%)	22 (45)	‒
Death, n (%)	3 (6)	‒

Values are presented as mean and standard deviation (±SD) or median and interquartile range (Q1‒Q3). n, number; g, grams. Non-paired Student *t*-tests or Mann-Whitney.

a p < 0.05.

b p < 0.01.

c p < 0.001.

d p < 0.0001.

Although the sepsis group showed twice as many male newborns as compared to female newborns, there was no statistically significant difference between the groups. The detected difference in the age at sepsis evaluation between sepsis and control groups is due to the prolonged length of stay of the newborn with sepsis in the NICU.

Hematological and laboratory data on D0 from the sepsis group were characterized by higher immature neutrophil count and immature/total neutrophil ratios, CRP, and direct bilirubin in comparison to controls.

Blood culture yielded growth in 63.3% of newborns and showed a predominance of Gram-positive bacteria with 10 Coagulase-Negative Staphylococci (CNS): seven *Staphylococcus epidermidis*, two *S. hominis*, and one *S. haemolyticus*; in the other 3 cases, two *S. aureus*, and one *Enterococcus faecalis* were detected. The 11 Gram-negative isolated bacteria were: *Enterobacter cloacae* (4 cases)*, Escherichia coli* (3 cases), *Pseudomonas aeruginosa* (1 case), *Klebsiella oxytoca* (1 case), *Stenotrophomonas maltrophila* (1 case) and *Acinetobacter baumannii* (1 case); the 6 fungi isolated were *Candida parapsilosis* (3 cases), *Candida albicans* (2 cases) and *Candida guilliermondii* (1 case).

### Kinetics of cytokine and lipoprotein levels during the NICU stay

**Fig. 1:**
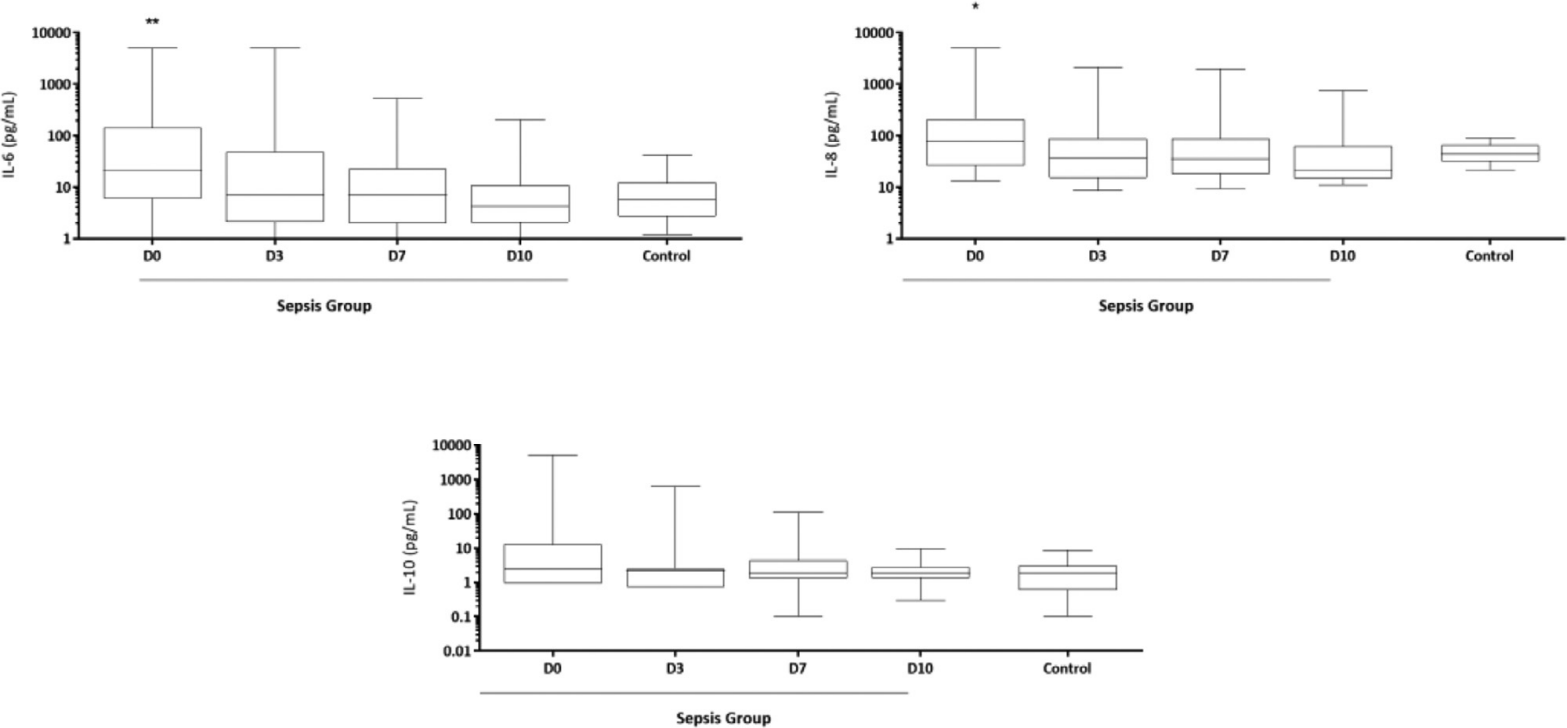
Time-serial measurements of serum IL-6, IL-8 and IL-10 concentrations from newborns with sepsis at the day of diagnosis (D0), and 3, 7 and 10 days (D3, D7 and D10) after initiation of treatment and from control group. *p < 0.05; **p < 0.01 vs. control group. Data were compared with control group by Student *t*-test.

IL-6 and IL-8 concentrations were significantly higher on D0 in septic newborns when compared to controls (Fig. 1). IL-1β, TNF-α and IL-12 concentrations were extremely low and did not show any differences in the analyses (data not shown).

Table 2 shows the results of CT, TG, lipoproteins, monocyte numbers and M/H ratios over time. No differences were found on D0 and D3 after sepsis had been diagnosed, but on D7, CT was found to be higher in septic newborns than in controls, and these higher levels were maintained on D10, which also presented higher TG, VLDLc, and non-HDL cholesterol concentrations than those from controls.

**Table 2:** Time-serial measurements of lipids and lipoproteins (mg/dL), monocyte numbers (10^6^/mL) and M/H ratios from newborns with sepsis at D0, D3, D7 and D10 post-diagnosis and from the control group.

	Sepsis (n = 49)				Controls (n = 17)
	D0 (n = 49)	D3 (n = 48)	D7 (n = 46)	D10 (n = 16)	
Total cholesterol	114.8 ± 31.7	123.4 ± 44	153 ± 96.3^a^	162 ± 59.1^a^	115.9 ± 36.9
Triglyceride	134.2 ± 63.9	155.3 ± 68.3	142.5 ± 82	174.4 ± 68.7^a^	124.6 ± 52.9
VLDLc	27.8 ± 14.9	28.1 ± 13.1	29.8 ± 24.5	36.2 ± 16.1^a^	22.4 ± 13.5
LDLc	53.9 ± 19	58.4 ± 21.5	80.7 ± 59.3	74.9 ± 30.4	57.9 ± 28.1
HDLc	29.1 ± 14.4^b^	35.7 ± 17.2	41.5 ± 23.4	46.7 ± 23.9	38.3 ± 17.6
Non-HDL cholesterol	81.7 ± 23.8	86.5 ± 31.3	109.8 ± 78.6	111.1 ± 44.2^a^	80.3 ± 33.6
Monocyte number	0.6 (0.3‒1.0)	0.5 (0.3‒0.8)	0.7 (0.6‒1.1)	0.7 (0.4‒1.4)	0.6 (0.3‒1.0)
Monocyte-to-HDL ratio	0.02 (0.01‒0.05)	0.02 (0.01‒0.03)	0.03 (0.01‒0.04)	0.02 (0.01‒0.06)	0.02 (0.01‒0.06)

Values are presented as mean and standard deviation (± SD) or median and interquartile range (Q1‒Q3). D, day; n, number. Data compared with control group by Student *t*-test.

a p < 0.05 vs. control group

b p = 0.07 vs. control group.

### Cytokine and lipoprotein levels and monocyte counts as predictors of septic shock

Of the 49 enrolled neonates, 22 (44.9%) progressed to septic shock and/or death (n = 3, 6%). In those subjects in whom septic shock developed, it occurred within a median of one day after study inclusion. All deaths were within 15 days after study inclusion: one on day 5, another death on day 6, and the last one on day 15 after diagnosis; all of them were unequivocally preceded by septic shock.

Data for the comparison between the newborns that developed septic shock and those who did not are summarized in Table 3. A higher number of male subjects progressed to shock, analyzed by the Chi-Square test (p = 0.017). Subjects who developed shock during the study period had higher CRP, IL-6, IL-8, IL-10, and lower TG, HDLc, monocyte numbers and M/H ratios on D0 than those from the non-shock group.

**Table 3:** Inflammatory markers, concentrations and Delta (Δ) lipids and lipoproteins, monocyte numbers and M/H ratios from septic shock and non-shock groups at D0.

	Septic shock (n = 22)	Non-shock (n = 27)
Sex (Male/Female)	19/3^c^	15/12
C-reactive-protein (mg/L)	55.8 (12.6‒92.9)^c^	6.2 (1.5‒20.6)
IL-6 (pg/mL)	130 (22‒2210)^d^	6.6 (4.8‒27.3)
IL-8 (pg/mL)	151 (94‒367)^c^	32.7 (18.1‒81.7)
IL-10 (pg/mL)	5.0 (1.0‒152)^b^	1.8 (0.6‒5.8)
Total Cholesterol (mg/dL)	105.2 ± 30.1	122.2 ± 31.5
a Δ Total Cholesterol (mg/dL)	-21.3 ± 31.6^b^	1,57 ± 29.1
Triglyceride (mg/dL)	109.0 ± 54.5^b^	154.6 ± 64.6
a Δ Triglyceride (mg/dL)	-39.9 ± 84.6	0.3 ± 69.0
VLDLc (mg/dL)	24.2 ± 12.8	30.2 ± 16.0
a Δ VLDLc (mg/dL)	-8.7 ± 13.2^b^	4.1 ± 14.8
LDLc (mg/dL)	47.3 ± 17.5	58.4 ± 18.9
a Δ LDLc (mg/dL)	-14.1 ± 13.5^b^	3.5 ± 25.5
HDLc (mg/dL)	28.9 ± 13.5	29.2 ± 15.4
a Δ HDLc (mg/dL)	-1.9 ± 9.0	-6.7 ± 9.8
Non-HDL cholesterol (mg/dL)	71.5 ± 25.3	88.7 ± 20.4
a Δ Non-HDL cholesterol (mg/dL)	-22.8 ± 17.6^d^	7.6 ± 26.4
Monocyte number (10^6^/mL)	0.3 (0.2‒0.5)^c^	0.8 (0.6‒1.6)
Monocyte-to-HDL Ratio	0.01 (0.007‒0.02)^d^	0.04 (0.02‒0.06)

Values are presented as mean and standard deviation (±SD) or median and interquartile range (Q1‒Q3). D, day; n, number.

a Delta (Δ) = D0 - D3; Data compared by Student *t*-test or Mann-Whitney or Person's Chi-Square test.

b p < 0.05;

c p < 0.01;

d p < 0.001.

Although septic neonates showed increasing lipid and lipoprotein levels between admission and D10 after diagnosis, without statistical significance for most of the parameters, as already shown in Table 2, when shock group vs. non-shock group were analyzed, decreasing levels of CT, VLDLc, LDLc and non-HDL cholesterol in septic shock group were observed when the differences between D0 and D3 (Δ lipoprotein = D0 - D3) for these variables were calculated (Table 3), which was not observed in the non-shock group, with the exception of HDLc.

The predictors for septic shock were studied by simple and multiple regression analysis. Total cholesterol and lipoproteins’ levels at admission did not achieve any association with the development of septic shock neither in simple or multiple (adjusted for gender) logistic regression, but TG, non-HDL cholesterol, and M/H ratio values, were significantly associated with septic shock development, even after correction by sex, although the 95% CI for TG was close to 1.0. Particularly, for M/H ratio, the Hosmer-Lemeshow test was non-significant only when one outlier sample was excluded (above percentile 98), revealing that the increase in 1 unit of M/H ratio, at admission, was associated with decreased odds of septic shock by 30%, and for the non-HDL cholesterol, by 4% (Table 4).

**Table 4:** Analysis of concentrations and Delta (Δ) lipids and lipoproteins and M/H ratio in septic patients at D0 as a risk factor for developing septic shock.

	Simple			Multiple (adjusted for gender)			
Variable	OR	95% CI	p^a^	OR	95% CI	Hosmer-Lemeshow test	p^a^
Total Cholesterol	0.98	(0.96; 1.00)	0.06	0.98	(0.95; 1.00)	0.125	0.063
bΔ Cholesterol	0.97	(0.95; 1.00)	0.021	0.96	(0.93; 0.99)	0.710	0.004
Triglyceride	0.99	(0.97; 1.00)	0.009	0.99	(0.97; 1.00)	0.721	0.014
bΔ Triglyceride	0.99	(0.98; 1.00)	0.096	0.99	(0.98; 1.00)	0.193	0.115
VLDLc	0.97	(0.92; 1.02)	0.182	0.96	(0.91; 1.01)	0.553	0.129
bΔ VLDLc	0.94	(0.88; 0.99)	0.011	0.91	(0.85; 0.98)	0.180	0.002
LDLc	0.97	(0.93; 1.00)	0.054	0.97	(0.93; 1.00)	0.476	0.064
bΔ LDLc	0.96	(0.93; 1.00)	0.024	0.96	(0.92; 1.00)	0.225	0.013
HDLc	1	(0.96; 1.04)	0.951	1.00	(0.95; 1.04)	0.125	0.895
bΔ HDLc	1.06	(0.98; 1.15)	0.132	1.05	(0.96; 1,14)	0.10	0.251
Non-HDL cholesterol	0.96	(0.93; 1.00)	0.015	0.96	(0.92; 0.99)	0.653	0.010
bΔ Non-HDL cholesterol	0.94	(0.90; 0.98)	<0.001	0.92	(0.87; 0.98)	0.542	<0.001
Monocyte-to-HDL Ratio	0.92	(0.85; 0.99)	0.001	0.70	(0.49; 0.99)^c^	0.343	<0.001

D, Day; OR, Odds Ratio; 95% CI, 95% Confidence Interval.

a Likelihood-ratio test.

b Delta (Δ) = D0 - D3.

c After exclusion of one outlier, Hosmer-Lemeshow test did not show statistically significant difference.

In addition, Table 4 also shows that increasing levels of CT, VLDLc, LDLc and non-HDL cholesterol from D0 to D3 (Δ lipid variables) were protective for septic shock development, after sex adjustment, although as for TG concentration at admission, the 95% CI of Δ LDLc also approached 1.0.

**Fig. 2:**
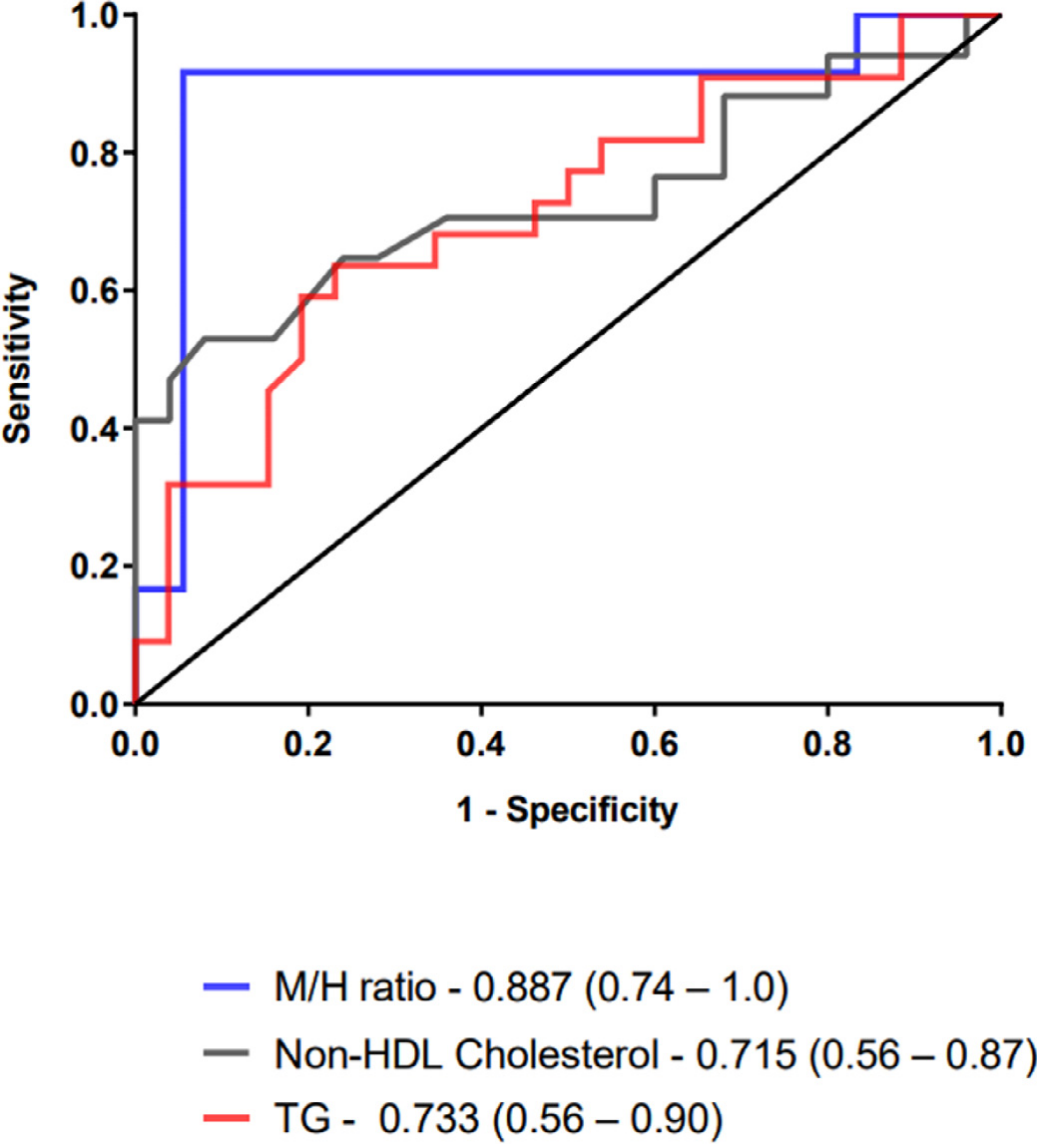
Receiver-Operating Characteristic (ROC) curves and their AUC values (95% CI) for M/H ratio, TG, non-HDL cholesterol for prediction of septic shock. AUC, Area Under the Curve; CI, Confidence Interval 95%.

The usefulness of TG, non-HDL cholesterol and M/H ratios as severity biomarkers was tested through Receiver-Operating Characteristic (ROC) curve analysis of patients with septic shock versus those who did not develop shock (Fig. 2).

The Area Under ROC Curve (AUC) of the M/H ratio was 0.887 (95% CI of 73.5% to 100%), and the cutoff value of < 17, defined by Youden index J, revealed a sensitivity of 91.7% (61.5%‒99.8%) and a specificity of 94.4% (72.7%‒99.9%) to predict septic shock in late-onset neonatal sepsis. However, AUC from TG and non-HDL cholesterol showed lower rates. The AUC of TG was 0.715 and using a cut-off of ≤114, the sensitivity and specificity were 66.7 (43.0‒85.4) and 76.9 (56.4‒91.0), respectively. The AUC of non-HDL cholesterol was 0.733 and using a cut-off of ≤65, the sensitivity and specificity were 52.9 (27.8‒77.0) and 92.0 (74.0‒99.0), respectively.

### Correlation analysis among lipoproteins and inflammatory mediators

To evaluate the impact of the inflammatory markers on the lipoprotein levels, correlation analyses on D0 were performed. Significant negative correlations were found among TG, VLDLc, LDLc and non-HDL cholesterol and the inflammatory markers, IL-6, IL-8, IL-10, and CRP, and among CT and HDLc and immature neutrophils and I/T ratio, as shown in Table 5.

**Table 5:** Correlations between IL-6, IL-8, IL-10, C-reactive-protein, immature neutrophils, I/T and lipoproteins at D0.

	IL-6	IL-8	IL-10	C-reactive-protein	Immature neutrophils	Immature/total neutrophil ratio
Total cholesterol	NS	NS	NS	NS	-0.327 (0.010)	-0.295 (0.020)
Triglyceride	NS	-0.409 (0.001)	NS	NS	NS	NS
VLDLc	NS	NS	-0.265 (0.045)	NS	NS	NS
LDLc	-0.354 (0.006)	NS	-0.293 (0.026)	-0.330 (0.006)	NS	NS
HDLc	NS	NS	NS	NS	-0.421 (0.001)	-0.348 (0.008)
Non-HDL cholesterol	-0.341 (0.009)	-0.279 (0.034)	-0.414 (0.001)	NS	NS	NS

D, Day; NS, Non-Significant.

## Discussion

It has been demonstrated that patients with sepsis have lower levels of cholesterol, including HDLc, LDLc, and Apo-A1, and higher levels of TG.^19^ In the present study, no differences were found in the lipoprotein levels on the day of diagnosis. Yildiz et al.^10^ reported significantly lower levels of CT, TG, HDLc and Apo-A and Apo-B in neonates with late-onset sepsis, but only Apo-A presented a relatively good sensitivity and specificity (73% and 97.2%, respectively) for sepsis diagnosis. Lipoproteins have been reported to be protective during sepsis, as they reduce the inflammatory response and mortality rates in experimental models.^6^^,^^20^

All lipoproteins play an important role in the binding and neutralization of LPS and lipoteichoic acid from Gram-negative and Gram-positive bacteria, however, it is clearly established that these antigens preferentially bind to HDLc particles.^21^ This neutralization consequently reduces macrophage activation, adhesion molecules expression, and the inflammatory cascade triggered by the Toll-like receptors present on these cells, interfering with the host's innate immune response possibly through the inhibition of NF-κβ pathway.^22^^,^^23^ The pleiotropic effects of HDLc include LPS neutralization, endothelial protection, and antioxidant and anti-apoptotic properties.^21^

Nevertheless, it has been suggested that during inflammation, Apo-A1 is replaced by Serum Amyloid A (SAA) in the HDLc particle, with a higher affinity for macrophages, which causes a redirection of HDLc from hepatocytes towards a macrophage scavenger pathway, promoting a more rapid turnover, along with reduced hepatic synthesis. This HDLc over-consumption and decline during sepsis could diminish its pleiotropic effects in the control of inflammation and promote an increased susceptibility to inflammatory stimuli, becoming a positive feedback loop that can ultimately lead to septic shock and death.^24^^,^^25^

During infection, the production of pro-inflammatory cytokines promptly induces lipolysis of adipose tissue and synthesis of liver fatty acids, causing an increase in TG and excessive production of VLDLc, while reducing CT, HDLc and LDLc serum levels, mainly due to the reduction in the cholesterol ester content in these lipoproteins.^26^ These changes have been described to be inversely related to the degree of inflammation measured by pro-inflammatory and anti-inflammatory cytokine levels (IL-6, IL-8 and IL-10) and by CRP levels, showing that the greater the degree of inflammation, the greater the changes in lipoprotein metabolism.^27^^,^^28^ These observations were also revealed in the present study by the inverse correlation indexes observed between the evaluated cytokines, PCR, immature neutrophils, and Immature-to-Total neutrophil ratio (I/T) with the lipoproteins.

Cholesterol and lipoprotein levels change rapidly over time in inflammation, especially in patients with severe infection or sepsis.^22^^,^^29^ In the present study it was observed an increase in TG, VLDLc, total cholesterol, and non-HDL cholesterol levels over time, reaching significantly higher concentrations on day 10 post-diagnosis. This observation agrees with others, who describe that hypertriglyceridemia is the most typical change in lipoprotein metabolism during infection and inflammation.^20^^,^^26^ TG metabolism is mediated by the cytokines TNF-α, IL-1, IL-6, and IFN-γ which promptly stimulate the synthesis of hepatic fatty acids, resulting in the increase in TG and in the production of TG-rich lipoproteins, while a decrease in the activity of the lipoprotein lipase, ultimately reducing the clearance of these lipoproteins.^19^^,^^30^

In contrast to previous studies, which performed comparisons between survivors and non-survivors, due to the small number of deaths, the present study was not designed to test a potential difference in mortality but rather to compare severe patients through the evaluation of septic shock development in the newborns. It has been argued that the relationship between lipoprotein levels and prognosis in septic adult patients is controversial, showing low HDLc and Apo-A1 levels in non-survivors^27^^,^^28^ or no significant HDLc differences between survivors and non-survivors, but significantly different TG levels instead.^8^

It has been reported that low HDLc levels at admission are a strong and independent prognostic factor of subsequent multiple organ dysfunction in adults.^7^ In the present study, we did observe lower HDLc and TG levels in newborns that developed septic shock, but without association with septic shock development in multiple logistic analyses. Moreover, it was also detected lower monocyte numbers, and M/H ratios in those patients, in addition to higher CRP, IL-6, IL-8, and IL-10 concentrations. It has been demonstrated that lower TG levels in adults with severe sepsis were associated with mortality on days 0 and 1 of admission^8^ and, in agreement, another study reported that increased TG serum levels were associated with decreased mortality rates.^31^ It has been suggested that increased total body fat oxidation and the clearance of TG in septic neonates may cause decreased TG levels, although the use of TG as an acute phase reactant for late-onset neonatal sepsis detection seemed less useful due to the low sensitivity and specificity.^10^

The present study evidenced an interesting finding that all lipoproteins diminished after 3 days from admission in the shock group, demonstrated by the delta calculations, while the non-shock group showed increasing levels on day 3, with the exception of HDLc. These decreasing levels were significant for CT, VLDLc, LDLc and non-HDL cholesterol, which also showed a positive predictive association with septic shock development. Nevertheless, this is probably a consequence of the severity of the sepsis, because all cases developed septic shock on the first day after admission.

A recent review presented several cardiovascular events in which an increased M/H ratio, due to an increased number of circulating monocytes and decreased HDLc levels, was reported to be positively correlated with poor outcomes^32^ agreeing with other studies on many different pathologies in adults.^33^^,^^34^ Nevertheless, in the present study, it was observed significantly reduced monocyte numbers in neonates who developed septic shock and, consequently, M/H ratio was significantly lower than in the non-shock group. Moreover, each unit elevation of M/H ratio at day 0 was associated with a decrease in risk of septic shock by 30%, even when corrected by gender. This result was consistent with those of the ROC curve analysis, which showed that M/H ratio at the admission of neonates for suspected sepsis is a reliable marker to early differentiate neonates who will develop septic shock, with the highest sensitivity (91.7%) and specificity (94.4%) among the analyzed parameters. To our knowledge, there are no studies in the literature that performed the analysis of M/H ratio in neonates with sepsis or septic shock.

It was recently reported in adult septic patients lower monocyte counts from day 3 to day 7 in non-survivors, and these lower monocyte counts were associated with the highest mortality, rate of bacteremia, and organ dysfunction, suggesting that monocyte count may serve as an independent predictor of 28-day mortality in septic patients.^35^

Another point to be considered is the nutritional status of the newborns during the NICU stay. Parenteral nutrition is widely used in preterm neonates in the initial period after birth, providing a relatively safe means of preventing nutrient deficits. Various lipid emulsions are available for use as part of parenteral nutrition, consisting of pure soybean oil, mixed lipid emulsions consisting of soybean oil plus Medium‐Chain Triglycerides (MCTs) and/or olive oil, and most recently, SMOFlipid (Fresenius Kabi, Germany), a multicomponent intravenous lipid emulsion containing 30% soybean oil, 30% MCTs, 25% olive oil, and 15% fish oil (referred to as “SMOF”).^36^ In the present series, the number of neonates in the shock group who did not receive SMOF or MCT (n = 4, 18.2%) did not differ from those in the non-shock group (n = 7, 25.9%).

Interestingly, there is some concern that soybean-based lipid emulsions could promote inflammation and suppress immune function, perhaps because of their high ω-6 PUFA and low ω-3 PUFA concentrations, leading several cell lines to apoptosis, including neonatal monocytes.^37^^,^^38^ PUFAs are susceptible to lipid peroxidation and the metabolites of this process are associated with increased oxidative stress, and increased intracellular levels of Reactive Oxygen Species (ROS) have been implicated in the regulation of Fas-mediated monocyte apoptosis.^39^ The majority of the neonates from the present study received SMOF containing olive oil and MCT to help reduce ω-6 PUFA content, and fish oil to provide the very long-chain ω-3 PUFAs, Docosahexaenoic Acid (DHA), and Eicosapentaenoic Acid (EPA). SMOF also contains α-tocopherol (vitamin E), at a higher level than those found in other lipid emulsions, which may help to reduce oxidative stress.^40^^,^^41^ In the pressent series, although a similar number of neonates received SMOF in both groups, a possible influence of parenteral nutrition on monocyte apoptosis cannot be ruled out.

## Conclusions

Reduced monocyte numbers and M/H ratios at the admission of neonates for suspected sepsis are associated with the development of septic shock, and these parameters were inversely correlated with inflammation markers, such as IL-6, IL-8 and IL-10. The present study revealed for the first time that non-HDL cholesterol and M/H ratios are predictive markers of septic shock, independent of gender, which may serve as additional clinically useful tools for identifying neonates who are at higher risk for adverse outcomes. Further prospective studies with a larger number of subjects are needed to address the prognostic capacity of M/H ratio in late-onset neonatal sepsis.

## Authors' contributions

Fernanda Andrade Macaferri da Fonseca: Formal analysis; investigation; methodology; writing-original draft.

Aline Paulino Espósito: Formal analysis; investigation; methodology.

Maria Helena Baptista da Silva: Investigation; formal analysis.

Valéria Sutti Nunes: Conceptualization; investigation; methodology; validation; supervision; writing-original draft.

Patricia Miralda Cazita: Conceptualization; investigation; methodology; writing-original draft.

Guilherme Silva Ferreira: Methodology; validation; formal analysis; writing-original draft.

Maria Esther Jurfest Rivero Ceccon: Conceptualization; investigation.

Werther Brunow de Carvalho: Supervision; Writing‐review & editing.

Magda Carneiro-Sampaio: Funding acquisition; resources; writing‐review & editing.

Patricia Palmeira: Conceptualization; funding acquisition; investigation; methodology; data curation; project administration; resources; writing‐review & editing.

## Funding

This work was supported by the FAPESP (Fundação de Amparo à Pesquisa do Estado de São Paulo) grants n° 2016/06887-5.

## Declaration of Competing Interest

The authors declare that they have no known competing financial interests or personal relationships that could have appeared to influence the work reported in this paper.
